# Comparison of two main treatment modalities for acute ankle sprain

**DOI:** 10.12669/pjms.316.8210

**Published:** 2015

**Authors:** Serkan Bilgic, Murat Durusu, Bahtiyar Aliyev, Serkan Akpancar, Omer Ersen, S.Mehmet Yasar, Sukru Ardic

**Affiliations:** 1 Serkan Bilgic, Department of Orthopaedics, Haydarpasa Military Training Hospital, Istanbul, Turkey; 2 Murat Durusu, Department of Emergency Medicine, Gulhane Military Medical Academy, Ankara Turkey; 3 Bahtiyar Aliyev, Department of Emergency Medicine, Gulhane Military Medical Academy, Ankara Turkey; 4 Serkan Akpancar, Department of Orthopaedics, Gulhane Military Medical Academy, Ankara Turkey; 5 Omer Ersen, Department of Orthopaedics, Maresal Cakmak Military Hospital, Erzurum, Turkey; 6 S.Mehmet Yasar, Department of Emergency Medicine, Gulhane Military Medical Academy, Ankara Turkey; 7 Sukru Ardic, Department of Emergency Medicine, Gulhane Military Medical Academy, Ankara Turkey

**Keywords:** Ankle injury, Elastic bandage, Splint

## Abstract

**Objective:**

Acute ankle sprains are one of the most common injuries in emergency departments. Immobilization is widely accepted as the basic treatment modality for acute ankle sprains; however, immobilization method remains controversial. In this study, we aimed to compare two treatment modalities: splint and elastic bandage for the management of acute ankle sprains.

**Methods:**

This prospective study was conducted in the emergency department. Fifty-one consecutive patients who were admitted to the emergency department owing to the complaint of ankle sprain and who were treated with an elastic bandage or a splint were included in the study. After bone injury was ruled out, treatment choice was left to the on-shift physicians’ discretion. The extent of edema was evaluated before and after the treatment by using a small, graduated container filled with warm water. Volume differences were calculated by immersing both lower extremities in a container filled to a constant level. Pain was evaluated using the visual analogue scale.

**Results:**

There were 25 patients in the elastic bandage group and 26 patients in the splint group. VAS scores of these groups before and after the treatment were similar. Although edema size before and after the treatment were similar between the groups, edema size reduction was significantly more in the elastic bandage group [p=0,025].

**Conclusions:**

This study showed that treatment of acute ankle sprains with an elastic bandage was more effective than splint in reducing edema. Therefore, an elastic bandage could be preferred over a splint for the treatment of acute ankle sprains.

## INTRODUCTION

Acute ankle injuries are one of the most common musculoskeletal injuries in emergency departments. The most common mechanism for such injuries is supination, combination of adduction and inversion of the plantar-flexed foot.^1^-^3^ The most common predisposing factor to an ankle sprain is a previous ankle sprain.^1^,^4^ Ankle injuries account for 3–5% and 2–6% of overall visits to emergency departments in Britain and the United States respectively.^5^-^7^

Although immobilization of the ankle is the basic treatment modality functional treatment methods that preserve range of motion of joints have been presented recently. In addition to the lack of evidence-based studies that compare treatment methods, no studies have definitely concluded the optimal functional treatment strategy for such injuries.

Studies that have investigated methods of treating ankle sprains have usually analyzed the time required to return to work or sports, to achieve complete range of motion, and resolve swelling and pain.^1^-^4^ In this study, we compared the effectiveness of two treatment modalities for the management of acute ankle sprain in terms of edema reduction and pain relief.

## METHODS

This prospective randomized study was conducted in the emergency department. Fifty-one consecutive patients who were admitted to the emergency department with the complaint of ankle sprain and who were treated with elastic bandage or splint were included in the study. Exclusion criteria were fracture on radiographs, previous ankle injury, and treatment methods other than bandage or splint. The Institutional Review Board of the relevant institution approved this study, and a written informed consent was obtained from each patient.

**Fig.1:**
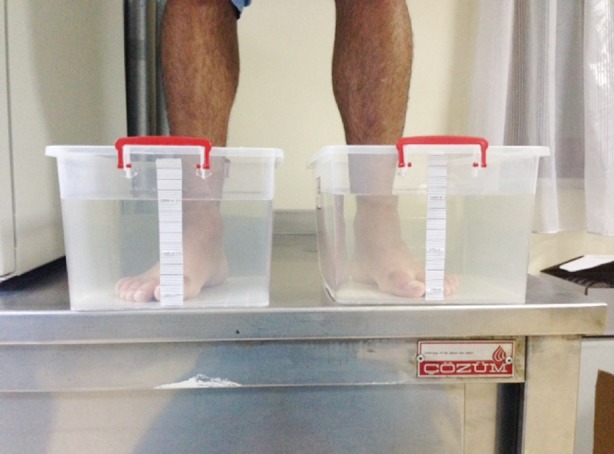
Measurement of edema by graduated containers.

After bone injury was ruled out on the basis of radiographs in the first evaluation, the choice of treatment was left to the on-shift physicians’ or resident doctors’ discretion. Before treatment, the extend of edema was evaluated using a small, graduated container filled with warm water, and these observations were compared to those of uninjured foot. Graduated containers were filled with 10 L of warm water and volume differences were calculated by immersing both lower extremities in the container (Fig.1). Pain was evaluated using the visual analogue Scale (VAS) score. All patients were administered paracetamol and were given standart advices standartly (Rest, ice compression, and elevation). Patients revisited the institution on seventh day for revaluation.

For statistical analyses, we used the SPSS software package (version 15.0, SPSS, Chicago, IL) and expressed categorical variables as percentages and continuous variables as mean ± standard deviation (SD) or median (quartiles). We used the Kolmogorov-Smirnov test to evaluate whether the distribution of continuous variables was normal. For parameters that showed normal distribution we used the paired samples t-test and for parameters that did not show normal distribution, we used the Mann-Whitney U-test. We used the Wilcoxon test to analyze categorical variables. Statistical significance was set at p < 0.05.

## RESULTS

There were 25 patients (11 female, 14 male) in the elastic bandage group and 26 patients (11 female, 15 male) in the splint group. The average age was 26.24±8.41 in the elastic bandage group and 32.15±12.74 in the splint group. Age and gender distribution were similar between the groups. Pain scores decreased significantly in both groups after 7 days of treatment however, the differences between their VAS scores were not significant (Table-I).

**Table-I:** Age gender distribution and Pain scores of the groups.

	Age	Gender F/M	Affected side R/L
Bandage	26.24±8.41	11/14	12/13
Splint	32.15±12.74	11/15	13/13

Pain score				
	Before	After	Difference	P
Bandage	7.08±1.98	0.88±0.60	6.20±2.04	<0.001
Splint	6.50±2.19	1.15±1.22	5.34±2.13	<0.001
P	0.40	0.80	0.12	

The extend of edema before and after treatment was similar in both groups. Both techniques reduced the extend of edema but reduction of edema had resolved was more significantly in the elastic bandage group than in the ‘splint group (p=0,025) (Table-II).

**Table-II:** Edema evaluations of the groups.

	Edema			
	Size differences (before)	Size differences(after)	Reduction of edema size	P
Bandage	199.20±288.77	99.20±273.20	100.00±40.82	<0.001
Splint	121.15±120.14	39.04±120.96	82.11±61.51	<0.001
P	0.47	0.95	0.025	

## DISCUSSION

Ankle sprains are very common injuries that are initially treated in the emergency departments. Most common ankle injuries are sports related but they also occur during via accidents in daily activities. These injuries cause disabilities and interfere in activities of daily living. Although ankle sprains are frequent, there is no clinical consensus among physicians about the management of ankle sprains and there is still no gold standard treatment method for ankle sprains yet.

The current concensus in the treatment of acute ankle sprains for adults indicates that functional treatment options such as taping or elastic bandage is superior to rigid treatment such as external ankle supports. However, there are insufficient results of studies that have compared external support and pure functional treatment. Moreover, current comparisons of treatment choices for ankle sprain have shown inconsistent results.^8^-^11^ In the literature, functional treatment methods and immobilization have been compared in the context of pain, range of motion, patient satisfaction, complications and swelling. However, the extent to which edema reduces with treatment has not been clearly studied. Unlike that in previous studies, in the current study, the extent to which edema reduced was evaluated by measuring the volume of water displaced by the injured foot compared to that displaced by the uninjured foot. The edema size reduced to a significantly greater extent in the elastic bandage group than in the splint group.

There are several scales for evaluating pain related to ankle sprains. Karlsson and Peterson created a scoring scale in 1991 to evaluate ankle joint function.^12^ Another scale is the Foot and Ankle Outcome Score that determines the pain related to ankle sprains.^13^ Although pain is one of the parameters in these scales, they are complex scales and not all clinicians in the emergency department are familiar with these. In this study, we used the VAS because of its easy applicability. VAS scores of both groups decreased and we found no difference in the VAS scores between groups.

Several variables such as age, gender, pain, swelling, previous injuries, complications, affect the outcomes of the treatment. Boyce et al. used the Karlsson Scoring Scale for evaluating primary outcome, Foot and Ankle Outcome Score for evaluating secondary outcome, and VAS score for evaluating pain.^11^ Owing to the differences in results of the functional treatments, consensus on the optimal treatment has not been reached.^2^,^14^ We believe that, owing to the multiple variables that affect outcome, it is difficult to determine the effect of each variable. Therefore, currently, we compared the results of two variables (reduction in edema and pain) between two treatment groups.

### This study is limited because of several factors

The small sample size of the study is the most important limitation. However, it is difficult to perform a prospective study on patients in a busy emergency department. Despite the frequency of presentation, several patients were excluded from the study because of the treatment used by on-shift physician or resident doctors. The study could not be blinded to the authors because of physicians had to treat patients directly.

## CONCLUSION

The results obtained from this study suggest that the use of an elastic bandage for functional treatment of acute ankle sprains a better preserves joint motion and reduces the extent of edema size than a splint, after 7 days of treatment. There is no significant difference in pain scores between the groups. Although this is a simple study with few variables, it provides valuable outcomes.
